# Human Placental Intracellular Cholesterol Transport: A Focus on Lysosomal and Mitochondrial Dysfunction and Oxidative Stress

**DOI:** 10.3390/antiox11030500

**Published:** 2022-03-04

**Authors:** Maria Jose Yañez, Andrea Leiva

**Affiliations:** School of Medical Technology, Health Sciences Faculty, Universidad San Sebastian, Providencia 7510157, Chile; maria.yanez@uss.cl

**Keywords:** placenta, cholesterol traffic, lysosome, mitochondria, oxidative stress

## Abstract

The placenta participates in cholesterol biosynthesis and metabolism and regulates exchange between the maternal and fetal compartments. The fetus has high cholesterol requirements, and it is taken up and synthesized at elevated rates during pregnancy. In placental cells, the major source of cholesterol is the internalization of lipoprotein particles from maternal circulation by mechanisms that are not fully understood. As in hepatocytes, syncytiotrophoblast uptake of lipoprotein cholesterol involves lipoprotein receptors such as low-density lipoprotein receptor (LDLR) and scavenger receptor class B type I (SR-BI). Efflux outside the cells requires proteins such as the ATP-binding cassette (ABC) transporters ABCA1 and ABCG1. However, mechanisms associated with intracellular traffic of cholesterol in syncytiotrophoblasts are mostly unknown. In hepatocytes, uptaken cholesterol is transported to acidic late endosomes (LE) and lysosomes (LY). Proteins such as Niemann–Pick type C 1 (NPC1), NPC2, and StAR related lipid transfer domain containing 3 (STARD3) are required for cholesterol exit from the LE/LY. These proteins transfer cholesterol from the lumen of the LE/LY into the LE/LY-limiting membrane and then export it to the endoplasmic reticulum, mitochondria, or plasma membrane. Although the production, metabolism, and transport of cholesterol in placental cells are well explored, there is little information on the role of proteins related to intracellular cholesterol traffic in placental cells during physiological or pathological pregnancies. Such studies would be relevant for understanding fetal and placental cholesterol management. Oxidative stress, induced by generating excess reactive oxygen species (ROS), plays a critical role in regulating various cellular and biological functions and has emerged as a critical common mechanism after lysosomal and mitochondrial dysfunction. This review discusses the role of cholesterol, lysosomal and mitochondrial dysfunction, and ROS in the development and progression of hypercholesterolemic pregnancies.

## 1. Introduction

The placenta is a large organ that develops during pregnancy, enabling optimal fetal growth. It can adapt to diverse external factors both structurally and functionally. In adaptation problems during placenta development, fetal survival or fetal growth will be in danger, and diseases could develop [1]. In addition, problems in the correct development of the placenta contribute to different diseases such as preeclampsia, which predispose the mother to lifelong diseases [2].

The different essential functions of the placenta include biosynthesis, metabolism, and transport of cholesterol, glucocorticoids, and sex hormones.

This article compares the hepatic and placental cellular management of cholesterol under physiological conditions and provides an overview of the changes observed due to preeclampsia and maternal hypercholesterolemic pregnancies. We will also discuss the role of cholesterol accumulation in syncytiotrophoblasts due to maternal hypercholesterolemia resulting from lysosomal and mitochondrial dysfunction and oxidative stress.

## 2. Cholesterol

Cholesterol is a sterol that was first isolated from gallstones. It is biosynthesized by all animal cells and is an essential structural component of cell membranes in mammals [3]. Cholesterol serves as a precursor for the biosynthesis of bile acid, vitamin D, and sex hormones such as testosterone, estradiol, androsterone, progesterone, and adrenocortical hormones such as aldosterone and cortisone. It is also required to form lipid domains involved in endocytosis and cell signaling. Cholesterol plays an important role in various homeostatic systems [4,5].

In physiological conditions, cellular cholesterol homeostasis includes tightly regulated processes. The body has two major sources of available cholesterol: (i) intestinal absorption of dietary and biliary cholesterol; and (ii) cholesterol biosynthesis in various tissues, predominantly in the liver and intestine. There are two main pathways for cholesterol excretion: (i) cholesterol excretion through the gastrointestinal tract and skin; and (ii) converting cholesterol to other compounds such as bile acids and steroid hormones [6]. It is important that the total cholesterol pool can be kept constant, since the total cholesterol input into the body must equal the total output in the steady state, to prevent the accumulation of excess cholesterol in the body [6]. High cholesterol biosynthesis in the liver leads to the synthesis of very-low-density lipoprotein (VLDL) secreted into plasma, increasing total plasma levels and low-density lipoprotein (LDL) cholesterol concentrations. Additionally, increased quantities of dietary cholesterol increase total plasma and LDL cholesterol levels in most individuals, which is an important risk factor for developing cardiovascular diseases in humans and laboratory animals [6,7]. Total and LDL cholesterol levels in plasma also fluctuate during physiological conditions such as pregnancy. This increase in lipid levels is known as maternal physiological hypercholesterolemia (MPH), a biological response to increased fetal demands. A significant number of pregnant women develop maternal supraphysiological hypercholesterolemia (MSPH). This condition is characterized by higher LDL levels than MPH and is associated with fetal and placental oxidative stress, endothelial dysfunction, and early fetal atherosclerotic lesions [8,9].

Given the complex regulation and diverse functions attributable to sterols, it is perhaps not surprising that inherited defects of genes involved in cholesterol metabolism or changes in the function of proteins required for proper homeostasis led to diverse metabolic alterations [10]. Before discussing this topic in further depth, we will first address aspects of cholesterol homeostasis to provide a framework for understanding the consequences of cholesterol metabolism/transport defects in humans, particularly in the vasculature of the placenta and the fetus from MSPH pregnancies.

## 3. Endosomal Cholesterol Transport

Mammalian liver cells contain several types of binding sites for plasma lipoproteins. Most cholesterol entering the cells is taken up by receptor-mediated uptake from lipoproteins. The core of lipoprotein particles is composed of triglycerides and cholesterol esters (i.e., fatty acylated cholesterol), while phospholipids and free cholesterol cover the particle surface. Endocytic circuits harbor substantial amounts of cholesterol that they acquire not only from lipoprotein uptake but also via membrane recycling and nanovesicular equilibration [11].

Cholesterol import from LDL and high-density lipoprotein (HDL) occurs via LDL-receptor-mediated uptake (LDLR) and HDL-mediated reverse transport via the scavenger receptor SR-BI, respectively. Additional receptors can mediate LDL and modify LDL uptake [12,13]; however, we will focus exclusively on the LDLR and SR-BI pathways in this review.

Lipoproteins are internalized via clathrin-coated pits into early endosomes. The receptor is recycled to the cell surface, and the LDL-particle is targeted for proteolytic and lipolytic degradation [14]. Alternatively, HDL particles can transfer cholesteryl esters to the plasma membrane without requiring endocytosis (selective lipid uptake) [15]. After internalization, the lipoprotein-cholesteryl esters are hydrolyzed. The enzyme responsible for the hydrolysis of cholesterol esters is lysosomal acid lipase. This lipase is present in lysosomes and earlier endocytic compartments [16]. This suggests that particle breakdown is initiated rapidly after internalization. The details of how cholesterol is trafficked within cells and how it leaves the lysosome remain subjects of investigation [17].

**Figure 1:**
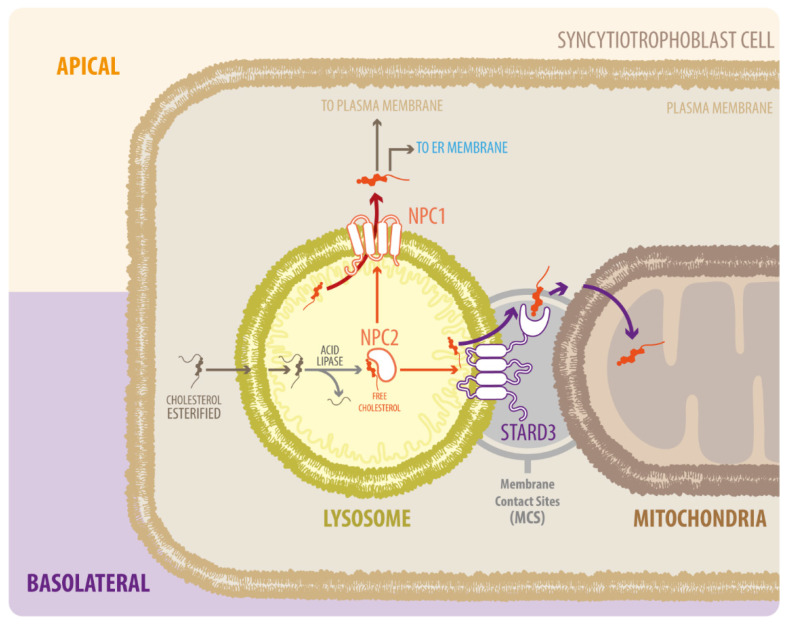
Diagram of NPC1, NPC2, and STARD3 proteins in LDL-derived cholesterol transport. Cholesterol from the endocytic pathway is hydrolyzed in the lysosome by acid lipase to form free cholesterol, which binds to NPC2 and is delivered to NPC1. NPC1 takes the cholesterol out of the lysosome via glycocalyx molecules on the internal surface of the lysosomal membrane. Cholesterol then moves to the endoplasmic reticulum (ER) and is distributed to the rest of the cell. Alternatively, NPC2 can transfer cholesterol from LE/LY directly to STARD3, which mediates transport to mitochondria; however, the exact mechanism by which this occurs is unknown.

After the action of lipase, free cholesterol appears in the late endosomes/lysosomes (LE/LY) [16]. Free cholesterol is transported across the cell to metabolically active pools or membranes via the proteins Niemann–Pick type C 1 (NPC1) and C2 (NPC2) or other less well-defined sterol carrier proteins [18]. NPC2 first binds to the hydrophobic side chain of unesterified cholesterol and transfers the molecule to the N-terminal domain of NPC1, which inserts cholesterol into the lysosomal membrane [19] (Figure 1). In cells affected by the NPC1 mutation and reduced NPC1 function, cholesterol transport from the late endosomes to various destinations, including the plasma membrane, is defective [20]. Impairment of the lysosomal cholesterol export pathway, mediated by NPC1 and NPC2 proteins, leads to cholesterol build-up and organelle and lysosomal dysfunction [17]. Although NPC1 and NPC2 participate in the entry and progress of cholesterol in the LE/LY system, functional loss of these proteins differentially affects different organelles. For example, several mitochondrial properties such as adenosine triphosphate (ATP) production, oxidative stress, and possible mitophagy are altered by NPC1 deficiency [21,22,23], probably due to increased mitochondrial cholesterol [24].

In contrast to NPC1 deficiency, the movement of endosomal cholesterol to the mitochondria is interrupted by NPC2 deficiency [25]. NPC2 mutants that bind cholesterol but cannot transfer cholesterol to NPC1 can restore cholesterol trafficking to the mitochondria in NPC2-deficient cells [25]. A possible explanation for this difference between NPC1 and NPC2 is that NPC2 transfers cholesterol from the LE/LY lumen directly to the perimeter membrane of LE/LY, as well as to other transmembrane proteins such as the StAR related lipid transfer domain containing 3 (STARD3) [26] (Figure 1). Several studies showed that STARD3 contributes to the growth of HER2-positive cancer cells, but given that both proteins do not directly interact, the molecular mechanism remains unclear [27]. Over the last years, the basic function of STARD3 has been clarified as a cholesterol transporter working at contact sites between endosomes and the mitochondria [28,29] or endoplasmic reticulum (ER) [30,31]. Remarkably, the proteins involved in intracellular cholesterol traffic have mostly been described in liver-derived cells. However, its role in other cells relevant for cholesterol metabolism, such as the placental cells, has been neglected.

To eliminate cholesterol, hepatocytes, and other cell types, including placental cells, secrete cholesterol to extracellular lipid acceptors such as lipoproteins, a process known as cholesterol efflux [30,31,32]. In hepatocytes, cholesterol efflux mechanisms include passive diffusion and active pathways mediated by ABCA1, ABCG1, and SR-BI. Several factors influence cholesterol efflux efficiency, including cellular cholesterol status, lipid transporter activity, and the nature of extracellular acceptors. In hepatocytes, ABCA1 and ABCG1 are important for eliminating cholesterol from cells and tissues and HDL biogenesis. ABCA1 stimulates cholesterol efflux to lipid-free apolipoproteins, predominantly to ApoA-I and ApoE. In contrast, ABCG1 promotes the efflux of cholesterol and oxysterols to pre-mature HDL. SR-BI can mediate cholesterol efflux from peripheral cells to HDL, but not to lipid-free ApoA-I [32]. Although most events related to these phenomena remain unclear, in the placenta, endothelial cells and syncytiotrophoblasts express the LDL receptor (LDLR), SR-BI, ABCA1, and ABCG1, which mediate cholesterol uptake and its release from cells to acceptors such as ApoA-I (via ABCA1) or HDL (via ABCG1) [33,34].

The placental syncytiotrophoblast has several active transporters on the apical and basal side that may regulate fetal transport. Several ABC transporters are expressed in the human placenta, where they play a role in transporting endogenous and exogenous compounds.

Previous studies by our group suggest that the ABCG1 transporter is mainly localized on the basolateral side of the trophoblast layer [35,36], suggesting a possible role for cholesterol export to fetal circulation. Interestingly, ABCG1 was reduced in MSPH compared to MPH. Regarding ABCG5 and ABCG8, the placental expression levels of these transporters are low, and there is little information about their localization and functional relevance [37].

## 4. Association of Abnormal Cholesterol Transport with Diseases

Among the approximately 7000 inborn metabolic errors, there is a family of diseases that result from defects in genes involved in sterol metabolism [10,38]. For example, a genetic disorder caused by a defect on chromosome 19 results in continued cholesterol production despite excess cholesterol in the blood (lack of uptake by the LDL receptor), which may cause familial hypercholesterolemia [39]. Many of these syndromes have corresponding mouse models; some are spontaneous mutants, while others have been generated by genetic manipulation. All these disorders have common phenotypes suggesting common pathological mechanisms.

If we focus on cholesterol trafficking disorders, a study by Vanier et al. [40] first reported the potential role of pathological cholesterol transport on two lysosomal storage diseases (LSDs); Niemann–Pick type C 1 (NPC1), and Niemann–Pick type C 2 (NPC2). Both diseases involve pathological storage of lipids (cholesterol and sphingolipids) in the CNS and peripheral tissues, and cholesterol is the main lipid involved in peripheral pathology [40]. NPC1 or NPC2 deficiency causes accumulation (storage) of unesterified cholesterol in LE/LY and prevents delivery to the ER and mitochondria. This cholesterol accumulation in LE/LY disrupts the expression of genes involved in cholesterol homeostasis, such as SREBP-dependent gene expression, promotes liver X receptor (LXR)-mediated responses, and impairs oxysterol generation [41]. Therefore, NPC has features of excessive cholesterol storage and deficiency [42].

Additionally, Smith–Lemli–Opitz syndrome (SLOS) is characterized by abnormal 7-dehydrocholesterol (7DHC) accumulation. SLOS patient fibroblasts cultured in a lipid-depleted medium synthesize cholesterol but exhibit significant cholesterol trafficking defects and accumulate unesterified cholesterol in LE/LY [43]. Additionally, 7-DHC accumulation in SLOS leads to lysosomal storage of cholesterol, sphingomyelin, and multiple GSLs, all hallmarks of NPC disease [44].

Congenital cholesterol metabolism defects have provided many fundamental insights into normal cholesterol homeostasis and cell biology. They have traditionally been viewed as discreet diseases with unique phenotypes; however, NPC and SLOS involve NPC pathway inhibition. Platt et al. [44] showed that SLOS disease involves secondary inhibition of the NPC pathway, since cholesterol storage in the late endocytic compartment is a common feature of these diseases. However, if NPC pathways inhibition drives pathology in other diseases, approved NPC disease therapies could be used for diseases that currently lack effective treatments.

## 5. Endosomal Cholesterol Transport in the Syncytiotrophoblast

Several studies have shown that maternal cholesterol can be transported to the fetus. The human placenta needs more than 1 g of cholesterol per day to facilitate fetal growth [45], and the human placenta manufactures approximately 400 mg of sex steroids from cholesterol per day [46]. Cholesterol is essential for myelination, the sonic hedgehog signaling pathway and fetal nervous system patterning [47]; therefore, human pregnancy is characterized by maternal hyperlipidemia, especially during the last trimester [48]. High maternal estrogen concentrations and insulin resistance stimulate hepatic VLDL production and increase triglyceride and cholesterol concentrations, providing ample cholesterol fuel for the placental cells [49].

During human pregnancy, the fetal circulation is separated from maternal blood by the placental barrier, which plays an essential role in fetal development and health by tightly regulating molecular exchange between the mother and the fetus [50]. The placenta develops from the blastocyst’s outer layer, which forms the undifferentiated cytotrophoblast (CTB). The CTB gives rise to two villi; the floating villus, where CTBs fuse to form the multinuclear syncytiotrophoblast (STB), and the anchoring villus [51,52,53,54]. The STB acts as an exchange barrier with the maternal blood to ensure that nutrients, waste, and gases are exchanged with the fetal blood [55]. The anchoring villus mediates the placental attachment to the endometrium in the uterine wall and sustains fetal growth [51,52,53,54].

Extravillous trophoblast cells (EVTs) are invasive mesenchymal cells which function to establish critical tissue connection in the developing placental–uterine interface. These cells proliferate and migrate from the cytotrophoblast in the anchoring villi of the placenta and invade the maternal decidua and myometrium and can induce vascular remodeling. The remodeling of uterine spiral arteries by EVTs is fundamental for effective placentation and perfusion of the intervillous space [56]. EVT functions are affected by intrauterine microenvironmental factors, including oxygen tension and inflammatory mediators [57].

The STB is a polarized layer of cells. The apical side is in contact with the maternal blood, and the basal side is in contact with the extracellular matrix and the fetal endothelium. Therefore, the expression of receptor or transporter proteins on one side of the STB determines the directionality of the released molecules. Maternal-to-fetal cholesterol trafficking requires STB mediated cholesterol uptake from maternal LDL and HDL through the endocytic LDLR, and the HDL receptor SR-BI, respectively [58,59], and LDLR and SR-BI expression has been reported in both placental endothelial cells and the STB [60,61,62,63]. Additionally, LDL-specific binding sites have been shown in preparations of microvillous placental membranes, representing enriched apical STB plasma membranes, throughout pregnancy [64,65].

Furthermore, the expression of LDLR mRNA in STB increases with advancing pregnancy in baboons [66]. Finally, HDL binding sites and SR-BI have been identified on isolated STB placental microvillous and basal plasma membranes [67]. LDL- and HDL-cholesterol is absorbed by the placenta or trophoblast in rodents and humans [58,62,68,69]. After receptor-mediated uptake, LDL particles are transported via the endosome/lysosome pathway, where the cholesteryl ester is hydrolyzed and transported by NPC2 and NPC1. Burke et al. [68] showed that NPC1 is expressed in the human placenta, and in mouse models the amount of NPC1 mRNA decreased in the placentas of mice fed with cholesterol compared to controls [70]. Additionally, NPC1 protein expression decreases in hamsters fed excess cholesterol [68]. However, changes in NPC1 expression in humans have not been investigated to date. Additionally, there is no information on placental NPC2 expression, there is only one paper on STARD3 expression in human STB mitochondria, and the role of STARD3 in the human placenta is still unknown [71]. Placental expression of other transporters, such as NPC1L1, ABCA2, SCP-x and HSP60, have been reported in humans [68]. Cholesterol transport in the human placenta remains an enigma.

A summary of the published information on proteins involved in cholesterol transport and metabolism in different placental cell lines is presented in Table 1. These proteins have been described in cell lines such as BeWo, JAR, and Jeg-3 (model of term cytotrophoblast), Swan 71 and HTR-8 (first-trimester trophoblast), and in primary cultures of STB; however, their functions and regulations have not been extensively investigated (Table 1).

The efflux of cholesterol from the basal side of the STB is poorly understood. ABCA1 and ABCG1 are expressed in placental endothelial cells and STB and could mediate cholesterol efflux via ApoA-I (via ABCA1) or HDL (via ABCG1) [35,63]. In the STB, cholesterol is released to fetal endothelial cells and into the fetal circulation via cholesterol transporters and acceptors [34,86,87]. ApoB, the main LDL protein, is expressed in the STB [61], and lipoprotein particles containing ApoB and ApoA-I have been isolated from placental tissue [78]. Additionally, the term placenta secretes ApoB-containing lipoprotein particles [88], and polarized grown BeWo cells secrete ApoB from both their apical and basal surfaces [61].

These observations suggest that cholesterol transport in the human placenta is similar to other tissues involved in cholesterol metabolism (i.e., hepatocytes), where the abundance and function of lipoprotein receptors and cholesterol transporters are regulated by cholesterol levels [89].

**Figure 2:**
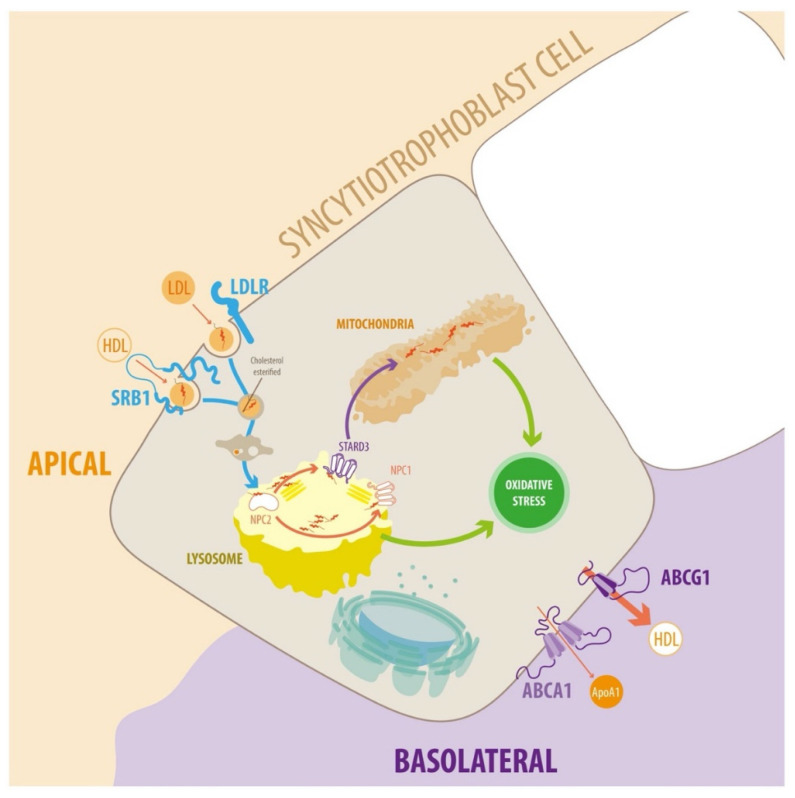
Diagram of endosomal cholesterol transport in the syncytiotrophoblast cells. In plasma, cholesterol is associated with different types of lipoprotein particles. LDL particles can interact with the plasma membrane of target cells via members of the LDLR family. The transfer of lipids between HDL and target cells is poorly understood. Several proteins and receptors bind HDL, such as SR-BI, which facilitates the uptake of cholesteryl esters. The receptor-ligand complexes dissociate after they enter acidic endosomal compartments. The receptors return to the plasma membrane, and lipoprotein particles enter the lysosomal pathway for degradation. Cholesterol incorporates into the endosomal/lysosomal membranes via NPC1 and NPC2. Cholesterol is transported to mitochondria for steroidogenesis via lipid transfer proteins such as STARD3. Cholesterol transport to other cellular targets, e.g., the plasma membrane, occurs via vesicular transport or cholesterol binding to various proteins. Alternatively, cholesterol can exit the STB via ABCA1 and ABCG1. ABCA1 stimulates cholesterol efflux to lipid-free apolipoproteins (predominantly ApoA-I, but also ApoE). Conversely, ABCG1 promotes the efflux of cholesterol and oxysterols to HDL. For detailed information, see text.

Placental intracellular cholesterol transport is still poorly understood (Figure 2) and requires further research.

## 6. Hypercholesterolemia in Pregnancy as a Predictor of Adverse Outcomes

Cholesterol is not measured in current obstetric practice. There are no reference ranges for circulating lipids during normal pregnancy because there is little evidence that elevated cholesterol levels are important. Normal pregnant women exhibit a physiological (i.e., normal) increase of around 30–50% in the total plasma cholesterol (TC) levels in a condition described in a previous section, known as the MPH or control state (C, TC levels lower or equal to 280 mg/dL) [90,91]. However, in some cases, TC levels increase far beyond this range, a condition known as maternal supraphysiological hypercholesterolemia (MSPH or hypercholesterolemic state, TC higher than 280 mg/dL) [90,92]. Circulating LDL levels increase during normal pregnancy, and this change is exaggerated in MSPH [17]. MSPH prevalence is ~30% [8,13,93], and although its etiology is still unknown, MSPH is associated with oxidative stress, endothelial cell dysfunction in placental vessels [13], and atherosclerosis in the fetal aorta [8,9]. Therefore, MSPH may be associated with cardiovascular disease in the offspring later in life, but the mechanisms are unknown.

MSPH is also associated with increased lipid peroxidation, levels of reactive oxygen species (ROS) and inflammation, in the placenta and fetus [9,94] (Figure 2). Conversely, in animal models, cholesterol-lowering or antioxidant treatment during pregnancy reduces atherogenesis, even though they do not lower maternal cholesterol levels [95,96,97].

Interestingly, MSPH neonatal TC levels are similar to those from normal pregnancies, suggesting some regulation of placental cholesterol traffic in MSPH pregnancies.

MSPH is associated with increased expression of genes involved in lipid metabolism in the placenta, exposing the fetus to an environment with a different lipid composition and promoting vascular alterations [98]. Additionally, increased maternal cholesterol and LDL (MPSH) levels decrease LDL receptor function and reduce SR-BI levels in the whole placenta and in primary human trophoblast cells (PHT) [36], suggesting that higher cholesterol concentrations in the maternal blood, regulate lipoprotein-derived cholesterol uptake. The same report also showed lower cholesterol efflux from the STB. The authors suggested placental cholesterol traffic is altered in MSPH pregnancies; however, there have not been any studies on modulating cholesterol levels during human pregnancy, even though the effect of this condition on the fetoplacental vasculature is well understood.

Cholesterol ester levels and free cholesterol levels are lower and higher in placental cells from MSPH pregnancies, respectively [36]. Free cholesterol usually indicates cell death [99,100,101]; however, its effects in PHT cells from MSPH is unknown. Additionally, the levels of HMG-CoA reductase (HMGCR), an enzyme that catalyzes the limiting step in the production of sterols, are lower in PHT from MSPH than controls, suggesting lower levels of endogenous cholesterol synthesis [36].

Fuenzalida et al. [36] showed that in MSPH, cholesterol transport and content in placental trophoblasts is altered, which could be associated with changes in placental-mediated maternal–fetal cholesterol transport. The abundance of LDLR and SR-BI was comparable between MSPH and MPH placentas. However, in PHT from MSPH, LDL and HDL uptake was lower than MPH, without changes in LDLR and reduced SR-BI levels. Regarding cholesterol efflux, in MSPH placentas, the abundance of cholesterol transporter ABCA1 was increased, while ABCG1 and SR-BI were reduced. In PHT from MSPH, cholesterol efflux to ApoA-I was increased and to HDL was reduced, along with reduced ABCG1 levels compared to MPH [36].

Therefore, MSPH may alter cholesterol trafficking and cholesterol content in placental trophoblasts to avoid exacerbated efflux of cholesterol to fetal circulation. Therefore, the absorption, transport, distribution, and supply of maternal lipids to the fetus are modulated by MSPH. However, how free cholesterol affects intracellular cholesterol pathways and cell viability is unknown.

There is little information about cholesterol biosynthesis in the placenta. Cholesterol synthesis and HMGCR activity in humans decrease as pregnancy progresses [102,103].

With advancing gestational age, novo cholesterol biosynthesis is markedly sup-pressed, with elevated concentrations of maternal serum-derived cholesterol, which re-places endogenously produced cholesterol as the major substrate of placental progesterone production in humans [59]. The addition of LDL to primary trophoblast cultures drastically suppresses the synthesis rates of cholesterol [69,104]. Nevertheless, when external LDL supply is reduced in vivo or in vitro, human trophoblasts produce sufficient progesterone due to endogenous cholesterol production [105]. As mentioned previously, human pregnancy is characterized by maternal hyperlipidemia, especially during the last trimester. Cholesterol concentrations provide ample cholesterol fuel for the STB [106]. There are estimates that synthesis rates of cholesterol in the term human placenta provides only 1–2% of the cholesterol required for progesterone biosynthesis [106]. Together, these data suggest feedback inhibition of maternal-derived cholesterol on endogenous cholesterol synthesis and cholesteryl ester formation in human STB.

Interestingly, maternal hypercholesterolemia does not change placental HMGCR protein levels nor free placental cholesterol or cholesteryl ester content [98]. Instead, it increases the placental expression of the transcription factor sterol regulatory element-binding protein 2 (SREBP-2) [98]. There is scarce information on the placental SREBP–SCAP–INSIG system [98]. As we mentioned, Fuenzalida et al. [36] observed that protein abundance of HMGCR was reduced in primary human trophoblast (PHT) from MSPH placentas, suggesting that cholesterol synthesis did not increase. In summary, human placental cholesterol synthesis regulation requires further characterization.

## 7. Lysosomal and Mitochondrial Dysfunction: Searching for Links in Hypercholesterolemic Pregnancies and Oxidative Stress

Lysosomes are important for various cell functions, including exocytosis, endocytosis/phagocytosis, autophagy, cell growth and death [39]. Many of these functions are mediated by acid hydrolase enzymes that degrade lipids, carbohydrates, proteins, and nucleic acids within the lysosome. Mitochondria are the intracellular organelles that produce adenosine triphosphate (ATP) via oxidative phosphorylation, regulate calcium homeostasis, and act as signaling platforms for several critical cell survival and apoptotic pathways [36].

Additionally, cholesterol circulation between late endosome/lysosomes (LE/LY) influences endomembrane traffic [41,42]. These compartments receive cholesterol from ingested plasma lipoproteins and the plasma membrane itself [43]. Cholesterol accumulation in the endosomal/lysosomal system impairs lysosomal function, and accumulated cholesterol “traps” the autophagy machinery, leading to impaired cellular homeostasis and function [44]. Additionally, increased mitochondrial cholesterol can impair mitochondrial function by reducing mitochondrial membrane fluidity [45] and decreasing ATP production [46,47] and mitochondrial glutathione (GSH) import [48,49]. The function of lysosomes and mitochondria in STB cells from MSPH placentas has not been evaluated yet despite increased free cholesterol levels.

Cholesterol is oxidized by enzymatic or ROS-mediated pathways when present in excess levels. Oxidized cholesterol increases in the cytoplasm [50] and disrupts cellular membranes, especially lysosomal and mitochondrial membranes [51]. Disrupted lysosomes are incapable of effectively removing ROS-damaged macromolecules [52], leading to a feedforward cycle of damage wherein ROS promotes the oxidation of cholesterol, disrupts lysosomal integrity, permeabilizes the mitochondrial membrane and finally kills the cell. Oxidative damage is a feature of many human diseases [53], and MSPH is associated with increased lipid peroxidation, oxidative stress and inflammation in the placenta and fetus [54,58,59]. However, the mechanisms driving increased ROS levels in MSPH are still unknown. MSPH placentas contain increased free cholesterol levels and ROS; therefore, we propose that LE/LY and mitochondrial function are compromised in MSPH placental cells.

As stated above, oxidative stress is an important factor in many complications during the second and third trimester of pregnancy. Preeclampsia, the most investigated pregnancy complication, develops in the second or third trimester and is characterized by maternal endothelial cell dysfunction resulting in systemic endovascular inflammation. This could cause symptoms such as proteinuria and hypertension. Inadequate extravillous trophoblast (EVT) invasion could result in an imbalance of oxidant/antioxidant activity when antioxidant capacity does not keep pace with increased oxygen tension, leading to a chronic state of oxidative stress. Early preeclampsia is often accompanied by fetal growth restriction [107,108], which is the second most studied pregnancy complication.

Watson et al. [109] showed that in the first trimester, the syncytiotrophoblast in the presence of high oxygen decreases microvilli at the surface and decreases mitochondria numbers without damaging cytotrophoblasts and stromal cells. Moreover, they demonstrated that the syncytiotrophoblast in early pregnancy expresses low antioxidant levels [110]. These results suggest that syncytiotrophoblasts can adapt to minimal increases in ROS by restoring the oxidant/antioxidant activity balance, which is seen in normal pregnancy.

## 8. Conclusions

Cholesterol accumulation and defects in cholesterol trafficking cause severe disease in humans and animal models. Cholesterol availability during pregnancy and its flux between the placenta and fetus are poorly described. Further investigation is needed to uncover the precise mechanisms of cholesterol trafficking between the mother and fetus. Additionally, the expression of proteins involved in cholesterol trafficking during pregnancy under normal and pathological conditions has barely been investigated.

We have established two main gaps in our knowledge of placental cholesterol trafficking: (1) The exact expression, localization, function, and regulation of key proteins required for proper intracellular cholesterol traffic (NPC1, NPC2, and STARD3, among others) are unknown. (2) It is unknown whether higher maternal levels of total and LDL cholesterol affect intracellular cholesterol trafficking in placental cells. However, high total and LDL cholesterol levels impair lysosomal and mitochondrial function and cell viability in other cell types (see Section 5 and Section 6). Therefore, the effect of chronic exposure to high cholesterol levels on placental cells requires further investigation.

## Figures and Tables

**Table 1:** Comparative table of proteins involved in cholesterol metabolism and transport.

Name	Source	Presence Proteins Cholesterol Metabolism	Reference
Swan 71	Primary first trimester	There is no information	There is no information
HTR-8	Primary first trimester	There is no information	There is no information
BeWo	Choriocarcinoma	VLDLR, LDLR, SR-BI, ABCA1, ABCG1, LRP1, ApoB	[33,36,61,72,73,74,75]
Jar	Choriocarcinoma	SR-BI	[74]
Jeg-3	Choriocarcinoma	SR-BI, STARD3, HSP60	[74,76]
STB	Placental	ApoB, ApoA-I, ApoE, LDLR, VLDLR, LRP1, LRP2, LRP8, ABCA1, ABCA2, ABCG1, SR-BI, NPC1, NPC1-Like1, NPC2, STARD3, SCP-x, HSP60	[35,36,61,67,68,77,78,79,80,81,82,83,84,85]

**Table legend:** A summary of the published information on proteins involved in the transport and metabolism of cholesterol in different placental cell lines. STB: syncytiotrophoblast; VLDLR: Very-low-density-lipoprotein receptor; LDLR: Low-density lipoprotein receptor; SR-BI: Scavenger receptor class B type 1; ABCA1: ATP-binding cassette transporter; ABCG1: ATP-binding cassette Subfamily G Member 1; LRP1: Low-density lipoprotein receptor-related protein 1; ApoB: Apolipoprotein B; STARD3: StAR Related Lipid Transfer Domain Containing 3; HSP60: Heat Shock Protein 60; ApoA-I: Apolipoprotein A1; ApoE: Apolipoprotein E; LRP2: Low-density lipoprotein receptor-related protein 2; LRP8: Low-density lipoprotein receptor-related protein 8; ABCA2: ATP-binding cassette transporter ABCA2; NPC1: Niemann–Pick type C 1; NPC1-Like1: Niemann–Pick type C 1-like1; NPC2: Niemann–Pick type C 2; SCP-x: Sterol carrier protein-x.

## Data Availability

The data presented in this study are available in review.

## Abbreviations

7DHC	7-dehydrocholesterol
ATP	Adenosine triphosphate
ApoA-I	Apolipoprotein A1
ApoB	Apolipoprotein B
ApoE	Apolipoprotein E
ABCG1	ATP Binding Cassette Subfamily G Member 1
ABCA1	ATP-binding cassette transporter
ABCA2	ATP-binding cassette transporter ABCA2
CTB	Cytotrophoblast
EVT	Extravillous trophoblast
GSH	Glutathione
HSP60	Heat Shock Protein 60
HDL	High-density lipoprotein
HMGCR	HMG-CoA reductase
INSIG	Insulin-induced Gene Protein
LE	Late endosomes
LXR	Liver X receptor
LDL	Low-density lipoprotein
LDLR	Low-density lipoprotein receptor
LRP1	Low-density lipoprotein receptor-related protein 1
LRP2	Low-density lipoprotein receptor-related protein 2
LRP8	Low-density lipoprotein receptor-related protein 8
LSDs	Lysosomal storage diseases
LY	Lysosomes
MPH	Maternal physiological hypercholesterolemia
MSPH	Maternal supraphysiological hypercholesterolemia
NPC1	Niemann–Pick type C 1
NPC2	Niemann–Pick type C 2
NPC1-Like1	Niemann–Pick type C 1-like1
PTH	Primary human trophoblast
ROS	Reactive Oxygen Species
SR-BI	Scavenger receptor class B type 1
SLOS	Smith-Lemli-Opitz syndrome
SCAP	SREBP cleavage-activating protein
STARD3	StAR Related Lipid Transfer Domain Containing 3
SCP-x	Sterol carrier protein-x
SREBP-2	Sterol regulatory element-binding protein 2
STB	Syncytiotrophoblast
TC	Total plasma cholesterol
VLDL	Very-low-density lipoprotein
VLDLR	Very-low-density-lipoprotein receptor
